# Macrophage-Based Microrobots for Anticancer Therapy: Recent Progress and Future Perspectives

**DOI:** 10.3390/biomimetics8070553

**Published:** 2023-11-18

**Authors:** Van Du Nguyen, Jong-Oh Park, Eunpyo Choi

**Affiliations:** 1 Robot Research Initiative, Chonnam National University, 77 Yongbong-ro, Buk-gu, Gwangju 61186, Republic of Korea; 2 Korea Institute of Medical Microrobotics, 43-26, Cheomdangwagi-ro 208-beon-gil, Buk-gu, Gwangju 61011, Republic of Korea; 3 School of Mechanical Engineering, Chonnam National University, 77 Yongbong-ro, Buk-gu, Gwangju 61186, Republic of Korea

**Keywords:** macrophage, cell microrobot, biomedical application, tumor targeting, anticancer, drug delivery, photothermal, magnetic

## Abstract

Macrophages, which are part of the mononuclear phagocytic system, possess sensory receptors that enable them to target cancer cells. In addition, they are able to engulf large amounts of particles through phagocytosis, suggesting a potential “Trojan horse” drug delivery approach to tumors by facilitating the engulfment of drug-hidden particles by macrophages. Recent research has focused on the development of macrophage-based microrobots for anticancer therapy, showing promising results and potential for clinical applications. In this review, we summarize the recent development of macrophage-based microrobot research for anticancer therapy. First, we discuss the types of macrophage cells used in the development of these microrobots, the common payloads they carry, and various targeting strategies utilized to guide the microrobots to cancer sites, such as biological, chemical, acoustic, and magnetic actuations. Subsequently, we analyze the applications of these microrobots in different cancer treatment modalities, including photothermal therapy, chemotherapy, immunotherapy, and various synergistic combination therapies. Finally, we present future outlooks for the development of macrophage-based microrobots.

## 1. Introduction

In recent years, robotic research groups worldwide have actively participated in the development of microrobots for efficient drug delivery in anticancer therapy applications. To enhance drug delivery, externally controlled microrobots have been developed. Artificial microrobots have been most commonly developed using various fabrication techniques. Especially with the fast development of 3D/4D printing technologies, these microrobots are easily fabricated with different sizes, shapes, and materials in a short amount of time and are capable of being mass produced [1,2,3,4,5,6]. However, these microrobots lack the intrinsic sensing ability needed to target and, more importantly, penetrate tumors since their actuations entirely rely on external sources, limiting the therapeutic efficacy and potential clinical applications. To address this limitation, biohybrid microrobots have recently been developed by biohybridizing microorganisms or biological cells with synthetic materials. Various techniques of fabricating biohybrid microrobots have been introduced in the literature [7,8,9]. Flagellum bacteria have been extensively used in the design and fabrication of these robots, as they can be bioconjugated by researchers with micro-to-nano-scale structures that can carry therapeutic agents [10,11,12,13,14,15,16,17,18,19]. These bacteria possess high levels of motility and some exhibit tumor-targeting abilities [14]. However, the use of bacteria is associated with several limitations, such as toxicity, ineffective bioconjugation, and small actuation forces [8,9]. Alternatively, macrophages can be used in macrophage-based microrobots to deliver the drugs to tumors [10,11,12]. Drugs or drug-loaded nanoparticles can easily be functionalized with macrophages through internalization via phagocytosis or surface conjugation with the cells [13]. Other important advantages of using macrophages for drug delivery to tumors include a reduced immune response as they are recognized as immune cells, tumor-homing ability due to their migration and chemotaxis properties [20], and their ability to traverse blood barriers, infiltrate tumors, and become tumor-associated macrophages, accounting for up to 80% of the tumor mass [14]. 

**Figure 1:**
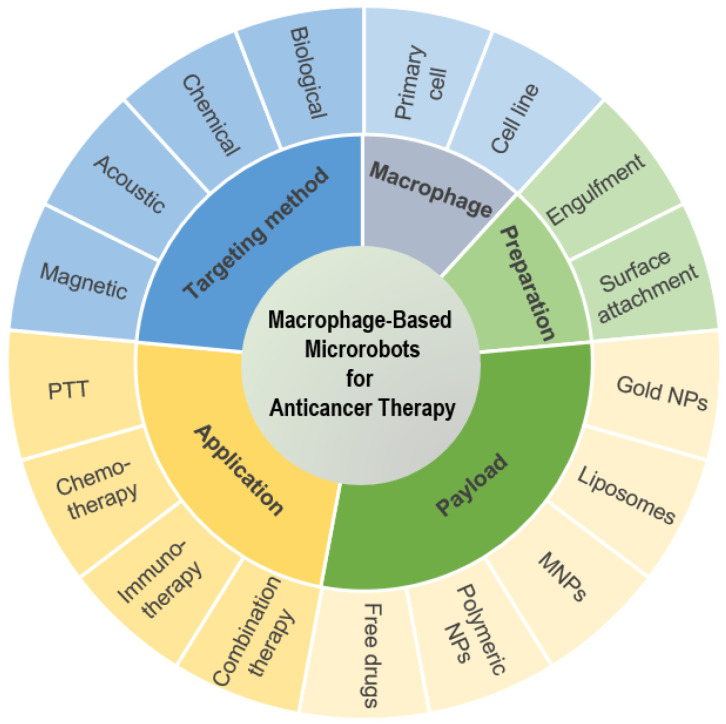
Review scope of macrophage-based microrobots for anticancer therapy: macrophage cell types, preparation methods, payloads, targeting methods, and their applications in anticancer therapy.

In this review, we summarize the recent developments in macrophage-based microrobot research for anticancer therapy. First, we focus on the popular types of macrophage cells used in the development of these microrobots, the types of payloads they carry, and the different targeting strategies employed to guide these microrobots to cancer sites, such as biological, chemical, acoustic, and magnetic actuation. Next, we analyze the applications of these microrobots in anticancer therapy (Figure 1). Finally, we present our outlook for the future development of these microrobots. To the best of our knowledge, this is the first comprehensive review of macrophage-based microrobots for anticancer therapy.

## 2. Preparation of Macrophage-Based Microrobots

### 2.1. Macrophage Cells

Macrophages belong to a group of mononuclear phagocytic cells of the innate immune system that plays an important role as the first line of defense against foreign objects, harmful pathogens, and tumorous cells [21,22,23]. Due to their characteristics, these cells can be utilized to create macrophage-based microrobots. To make such microrobots, commercial monocytes (precursors of macrophages, which become macrophages after migrating from capillaries to tumors) and macrophage-like commercial cell lines are commonly used to construct macrophage-based microrobots. These cells include the mouse cell lines RAW 264.7 [24,25,26,27,28] and J774A.1 [29,30], rat alveolar macrophages [31], and human original cells such as THP-1 [32,33]. However, to enhance the biocompatibility of these microrobots for in vivo use, primary macrophages are obtained both from animals, such as peritoneal [34], spleen-derived [29], and bone marrow cell-derived macrophages [35,36,37,38], and humans, such as human macrophages [39].

### 2.2. Preparation of the Microrobots

There are two main methods that are widely used to prepare microrobots. In the first method, which is used in a majority of related studies, the payloads are “eaten” or engulfed by macrophages when co-incubated in culture media for a certain period of time due to the strong phagocytosis ability of the macrophages [40]. In the second method, the payloads are chemically bound to the surface of the macrophage [41]. Mitragotri and colleagues introduced the attachment of disk-shaped backpacks (BPs) with a diameter of 7 μm and a thickness of 500 nm. They showed that the BPs were not phagocytosed by monocytes and were strongly attached to the cell surfaces due to their particular shape and flexibility. As a result, the BP-laden monocytes could hitchhike to the inflamed skin or lung in vivo [42]. In a recent study, Yang et al. prepared biotin-modified liposomes loaded with Dox and attached them to the surfaces of RAW 264.7 macrophages, which were modified with streptavidin-conjugated (polyethylene glycol) PEGylated lipids, via high-affinity biotin-streptavidin (MA-Lip). They showed that the MA-Lip infiltrated deeper, enhanced Dox accumulation, and increased the antitumor immune response [43]. Each method has its own advantages and disadvantages. While the first one provides a simple means for preparing a microrobot, the premature released drugs from the payloads may affect the functions of the macrophages upon their engulfment. In the second one, the preparation is more complicated because of the surface modification of the macrophages and the functionalization of the payloads. However, since no therapeutics are internalized into the macrophages, the cells will have a higher chance of survival when reaching the targeted sites. 

### 2.3. Payloads

Several materials have been adopted as payloads for macrophage-based microrobots to induce therapeutic effects. Depending on the specific applications or experimental settings, macrophages can engulf and transport multiple types of payloads.

#### 2.3.1. Gold-Based Nanoparticles

Gold nanoparticles are biocompatible and have a good ability to absorb near-infrared (NIR) light. In a pioneering work, Choi et al. used Au nanoshells phagocytosed by monocyte-derived macrophages to penetrate intratumorally into tumor spheroids and induce cell death in both the macrophages and tumor cells through photoinduction [39]. In addition, gold nanorods (AuNRs) have been widely used as payloads for macrophage-based microrobots due to their longitudinal surface plasmon resonance peak in the NIR window, allowing them to efficiently convert NIR light energy into heat [44]. Li et al. utilized RAW 264.7 macrophages that engulfed small AuNRs with a size of 7 nm, which showed high cell viability after engulfment. The AuNR-laden macrophages were found to enhance tumor coverage and improve phototherapy in vivo [45]. In another study, An et al. prepared macrophages loaded with AuNRs of different surface charges (cationic, neutral, anionic), showing promising photoacoustic imaging of tumor hypoxia and enhanced in vivo photothermal therapy of the tumor [24].

#### 2.3.2. Liposomes

Liposomes are vesicles consisting of lipid bilayers. They are biocompatible and biodegradable materials that can be loaded with both hydrophobic and hydrophilic therapeutics. By adjusting the lipid composition, liposomes can be designed to release therapeutics in a controlled manner. Based on these merits, liposomes are widely adopted as payloads for macrophage-based microrobots since they protect the macrophages from cell death and premature release of the payloads before reaching the targeted sites. Choi et al. prepared 150 nm liposomes loaded into peritoneal macrophages and observed high in vivo migration and positive therapeutic effects in an A549 tumor-bearing mouse after administering five doses [34]. Fujita and colleagues incorporated magnetic lipoplexes (SPION-incorporated cationic liposome/pDNA complexes) into RAW 264.7 macrophages. They showed that cytokine release was similar in engineered and pristine macrophages, but the production of nitric oxide was significantly enhanced in the engineered cells. In addition, under a magnetic field, the engineered cells exhibited strong attachment to a Caco-2 cell layer and the colon of mice, suggesting improved colonic delivery and potential therapy for colonic inflammation [46].

#### 2.3.3. Magnetic Nanoparticles (MNPs)

MNPs have been widely used in biomedical applications such as biomedical imaging agents due to their excellent biocompatibility and magnetic properties [47]. In addition, they have been used to enhance the functionality of the systems carrying them, specifically through their controllability via a magnetic field [27]. Recently, Li et al. modified MNPs by incorporating them with bioengineered bacterial outer membranes, generating biogenic macrophage-based microrobots (MΦ-OMV robots). These robots were able to be manipulated in vitro in a confined space, and in vivo in a mouse tumor model [48]. We used poly-(vinyl alcohol)-coated (PVA-coated) MNPs encapsulated in paclitaxel liposomes and engulfed by J774A.1 macrophages, enabling dual controllability of the macrophages through an external magnetic field and chemotaxis [49]. In addition, MNPs show responsiveness to near-infrared (NIR) light from a laser [50]. Therefore, they can be used as therapeutic agents that convert light energy into heat when irradiated using an NIR laser [51,52,53,54].

#### 2.3.4. Polymeric Nanoparticles

Biocompatible polymeric nanoparticles offer many advantages for drug delivery, such as nontoxicity and a prolonged controlled release of the encapsulated drugs. These properties make polymeric nanoparticles ideal candidates for encapsulation into macrophages since they can prolong the lifespan of macrophages due to their slow drug release rates. Therefore, many researchers utilize these nanoparticles as payloads for macrophage-based microrobots [32,35,55,56,57]. Xie et al. used biodegradable photoluminescent poly-(lactic acid) decorated with muramyl tripeptide, loaded with a drug (PLX4032), and engulfed the nanoparticle complex to macrophages. The engineered macrophages could carry the drug to the cancer cells via cell–cell binding. The authors proved that the system effectively killed the cancer cells [32]. Our group created a macrophage-based microrobot by utilizing the phagocytosis of poly-lactic-co-glycolic acid loaded with MNPs and docetaxel anticancer drugs that allowed for hybrid control of the microrobot and the ability to destroy cancer cells using the drug released from the microrobot [55]. Shi and coworkers internalized hyaluronic acid nanogels, prepared using a double-emulsion method, encapsulated with DOX and polypyrrole into RAW 264.7 macrophages to create macrophage-based microrobots (MAs-NGs). As a result, upon treatment with the MAs-NGs followed by laser irradiation, a subcutaneous cancer model was significantly inhibited [56].

#### 2.3.5. Free Drugs

The engulfment of free drugs into macrophages has also been studied in the literature. Fu et al. engineered RAW 264.7 macrophages with a high concentration of Dox solution (400 μg/mL, 1.8 mL for 1 million cells). As a result, a therapeutically meaningful amount (100 μg) of Dox was encapsulated in one million macrophages. In addition, the Dox-modified macrophages displayed good tumor-homing abilities and promising metastasis inhibition [58]. Guo et al. loaded Dox directly into M1 macrophages (M1–Dox) stimulated from RAW 264.7 cells. The authors reported that the engineered macrophages significantly enhanced tumor-homing ability by upregulating CCR2 and CCR4 compared to un-engineered cells. In addition, M1–Dox prevented tumor invasion induced via Dox. Moreover, compared with a commercial liposomal product (Lipo–Dox), M1–Dox showed a superior penetration and deeper accumulation within disseminated neoplastic lesions, leading to a critical reduction in metastatic tumors and an increase in the survival rate [28]. 

## 3. Targeting of Macrophage-Based Microrobots

### 3.1. Biological Targeting

**Figure 2:**
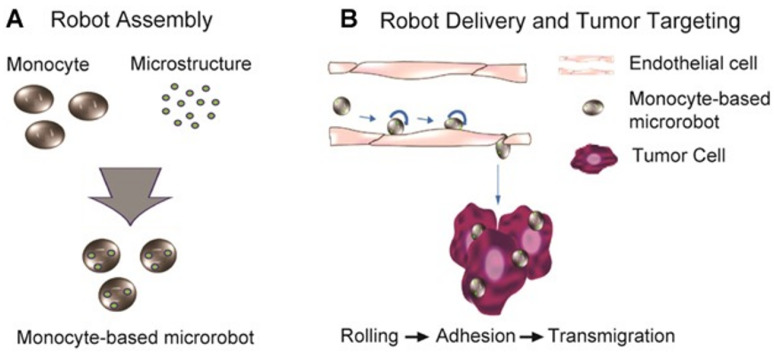
Monocyte-based microrobot with tumor-targeting properties. (**A**) Schematic representation of the fabrication of monocyte-based microrobots and (**B**) tumor-targeting concept of monocyte-based microrobots transmigrating over endothelial cells. Reprinted with permission from ref. [33]. Copyright 2014, Wiley Periodicals, Inc.

The homing of macrophage-based microrobots to the target sites is based on the intrinsic characteristics of the macrophages that possess sensory receptors capable of identifying foreign substances and detecting signs of inflammation within a living organism. Additionally, they exhibit self-actuating properties that allow them to migrate from the bloodstream to specific target locations within tissues in response to signals indicating infection or inflammation [33]. It is widely believed that activated macrophages can differentiate tumorous cells from normal cells by detecting the different compositions of the membranes of the targeted cells. These tumor-specific markers may include an elevated presence of phosphatidylserine. Moreover, additional recognition mechanisms may involve identifying changes in carbohydrate structures, known as glycosylation, on the surfaces of tumorous cells. Some tumor antigens, such as carcinoembryonic antigens and Tn antigens, are specific carbohydrate structures that can be detected by the lectin-like receptors expressed from macrophage cell membranes [59]. Park et al. fabricated a monocyte-based microrobot using the engulfment activity of THP-1 monocytes and tested their penetrating chemotactic motility toward various chemo-attractants prepared in a multi-layered cell migration chamber resembling a blood vessel barrier (Figure 2). The experiment results showed that the microrobots had a migration ability similar to that of the original macrophage [33]. Ren et al. loaded anti-inflammatory resveratrol and indocyanine green (ICG)-encapsulated octa-arginine-modified liposomes into macrophages derived from inflammatory monocytes isolated from peritoneal lavage. The engineered macrophages displayed a compelling tumor-targeting ability through their inflammatory tropism [60]. The advantages of biological targeting include biocompatible intrinsic power sources, the ability to combine sensing and targeting, and the appropriateness of fluids in physical environments [61]. The disadvantages are the small actuation force and weak targeting ability. Therefore, to enhance the targeting ability, other targeting strategies, which are discussed below, should be used in combination with the self-targeting ability of macrophages.

### 3.2. Chemical Targeting

Chemical targeting of microrobots is achieved through chemical reactions that generate bubbles to propel the microrobots. A typical reaction is as follows [62]:

H_2_O_2_ → H_2_O + O_2_ (1)

The propulsive mechanism relies on the catalytic decomposition of hydrogen peroxide (H_2_O_2_), which is available in specific environments or conditions (fuels), into water (H_2_O) and oxygen (O_2_) via platinum nanoparticles (Pt NPs) coating the inner surfaces of the microrobots or their carrying payloads. This process results in the generation, formation, and release of O_2_ bubbles from one end, initiating motion in the opposite direction [62].

Other chemical reactions could be as follows [63,64,65,66]:

Mg + H_2_O → Mg(OH)_2_ + H_2_ (2)

Mg + HCl → MgCl_2_ + H_2_ (3)

Mg + 2H^+^ → Mg^2+^ + H_2_ (4)

Zn + 2H^+^ → Zn^2+^ + H_2_ (5)

**Figure 3:**
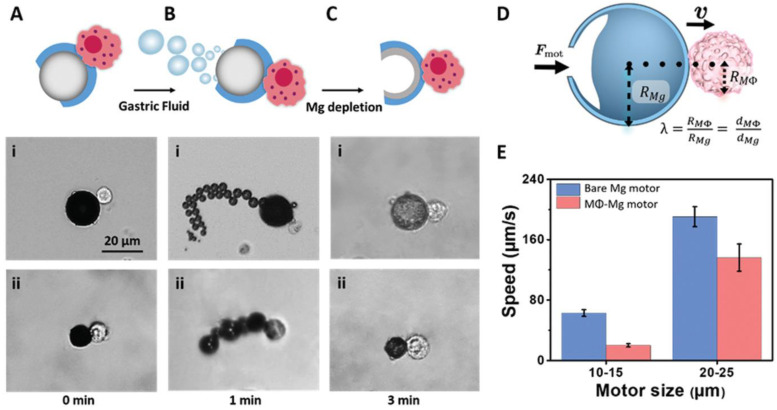
Propulsion characteristics of MΦ–Mg motors. (**A**) Schematic and microscopy images of a MΦ–Mg micromotor with motor diameters of: (i) 20–25 µm or (ii) 10–15 µm at time = 0 min. (**B**) Schematic and microscopy images of a MΦ–Mg micromotor with motor diameters of: (i) 20–25 µm or (ii) 10–15 µm propelling in gastric fluid solutions at time = 1 min. (**C**) Schematic of the MΦ–Mg shell (with partial Mg depletion) at time = 3 min, along with microscopy images of a MΦ–Mg shell corresponding to the two motor sizes (i, ii) at time = 3 min. (**D**) Modeling of the dynamics of a dimer where a MΦ of an effective radius RMΦ is attached to a Mg-based micromotor with a radius of RMg (following partial Mg depletion). (**E**) Comparison of the speed of 10–15 µm and 20–25 µm Mg motors and MΦ–Mg motors in the gastric fluid simulant (values defined at time = 1 min). Reprinted with permission from ref. [67]. Copyright 2019, WILEY-VCH Verlag GmbH & Co. KGaA, Weinheim, Germany.

In Equations (2)–(5) above, hydrogen bubbles are generated when the surfaces of the microrobots or their carrying payloads are composed of specific metals, such as zinc (Zn) or magnesium (Mg), to propel the microrobots. Regarding this targeting approach, Wang et al. has recently developed a hybrid macrophage microrobot (MΦ–Mg motor) by combining a J774A.1 macrophage with a Mg Janus microparticle coated with titanium dioxide (TiO_2_) and poly-(L-lysine) (PLL) (Figure 3). The PLL layer on the outer surface of the microparticle was used to facilitate the effective stable attachment between the microparticle and the macrophage through electrostatic interaction. In addition, the partially coated PLL layer also exposed the TiO_2_ layer to gastric fluid, propelling the microrobots. They showed that the microrobots could move at a relatively high velocity of 127.3 μm/s [67]. Most recently, Cai et al. prepared a twin-engine yeast microrobot (TBY robot) that that could be self-driving and self-adapting to autonomously approach the gastrointestinal inflammation site using an enzyme-driven engine and a macrophage bio-engine [68]. The TBY robot comprised a Janus distribution of glucose oxidase and catalase over the surfaces of yeast microcapsules. Therefore, in a glucose environment, the robot would produce a concentration gradient of glucose surrounding it, thus creating convective flows to actuate the robot undertaking the self-driving motion. The authors verified that the robots could achieve a maximum velocity of 8.9 μm/s and that they could propel themselves at a glucose concentration as low as 10 mM, which is much lower than the concentration in the lumen of humans and rat small intestines of 48 and 50 mM, respectively, thus proving the good applicability of the robots. After that, the robots were switched in situ to the macrophage engine in the Peyer’s patch and migrated intrinsically to inflammation sites. Using this approach, the drug concentration at the site increased roughly 1000-fold.

Although the induced propulsion velocity with the chemical actuating method was faster than other actuations, the locomotion seemed less directional and the targeting accuracy, quick action, and immediate feedback were insufficient [61]. In addition, the fuels required for the microrobots were highly toxic, limiting the applications of this targeting strategy [69,70]. 

### 3.3. Acoustic Targeting

**Figure 4:**
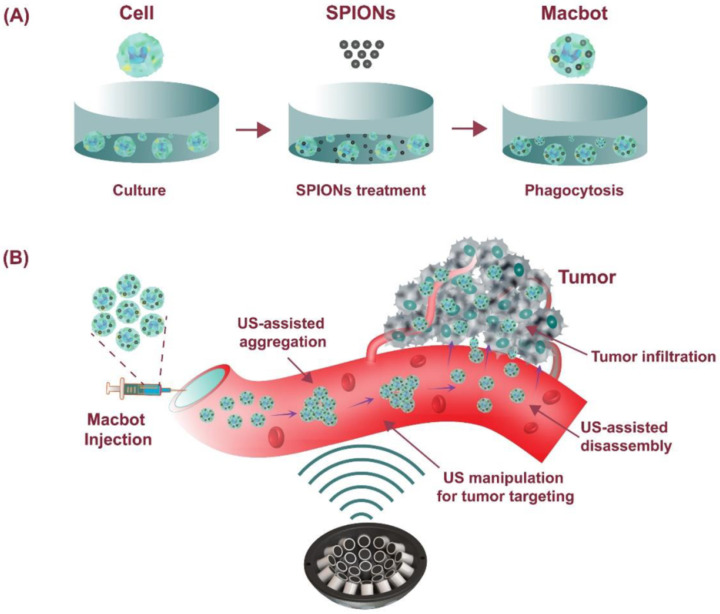
Schematic diagram showing the concept of using acoustically driven cell-based microrobots (Macbots) for targeted tumor therapy: (**A**) fabrication process of the Macbots; (**B**) working principle of the Macbots using an ultrasonic actuator system. Reprinted from ref. [80] under the terms of the CC-BY license.

Acoustic tweezers have been used for manipulating micro-nanoscale substances using physically induced effects exerted by acoustic waves. These waves generate acoustic radiation force when interacting with solids, liquids, and gasses, allowing for the capture of objects ranging from microscale to centimeter-scale sizes without physical contact [71]. These instruments have become versatile tools with applications in disease diagnosis, cellular manipulation [72,73], and various in vivo scenarios [71]. Acoustic tweezers can be categorized into three groups based on their operational principles: (i) standing waves, (ii) traveling waves, and (iii) acoustic streaming. The first two categories utilize external acoustic radiation forces for object manipulation, while acoustic streaming tweezers control object orientation through the flow of media. Acoustic tweezers based on standing waves employ interdigitated transducers and are typically utilized for separating and patterning particles and cells within channels [74]. In this configuration, a single acoustic beam is generated via the overlap of traveling waves at a single point. The location of this point can be changed by modulating the phase of each transducer, allowing for precise manipulation of targeted objects [75]. However, this setup is not suitable for in vivo environments as it relies on opposed transducer pairs that may not be applicable to patients depending on their treatment area. In contrast, traveling-wave tweezers have the capability to dynamically create pressure nodes in a three-dimensional space, either utilizing a single element or a multi-element setup, in real-time. In addition, traveling waves can induce the pressure field without the need for a reflector, making a single-sided array of transducers suitable for various in vivo applications [76]. When multiple traveling waves interfere at a single point, they collectively produce a single acoustic beam. Furthermore, the position of this point can be adjusted by modulating the phase of each transducer, enabling the precise manipulation of targeted particles into specific positions. Using traveling waves involves both passive and active control of the acoustic phase pattern. The passive approach involves physical control methods, including acoustic metamaterials and lenses, which necessitate redesigned structures to alter the phase pattern. Due to these limitations, this system requires a motorized stage for real-time object manipulation [77]. In this context, the active method serves as a suitable solution, enabling the adjustment of individual phase values through electronic control. Using this method, the trapping point can be repositioned in a three-dimensional space without the need for additional driving equipment. The active traveling approach employs various phase patterns to actuate objects, such as vortex, bottle, and twin trap [78]. Among these, the twin trap, characterized by the phase pattern of two cylindrical beams, is particularly effective, offering a large acoustic trapping force with a wide range of guiding angles [79]. Using this approach, our group developed an innovative acoustic manipulation system and designed macrophage-based microrobots, referred to as ‘Macbots’, for a potential actively targeted tumor therapy. The suggested acoustic tweezer comprises 30 ultrasonic transducers that operate at a frequency of 1 MHz, resulting in the generation of a twin-trap configuration at the trapping point. These Macbots were equipped with superparamagnetic iron oxide nanoparticles to function as carriers for drugs. When subjected to an acoustic field, a cluster of Macbots could be precisely maneuvered along a predefined path, allowing them to approach the target from various angles. In order to validate the fundamental aspects of this approach, we conducted an in vitro experiment focused on targeted tumor therapy (Figure 4). 

As a result, we were able to manipulate the cluster of microrobots to any point within a 4 × 4 × 4 mm region of interest, with a remarkable position error of less than 300 µm. Additionally, the Macbots exhibited the capability to rotate within the O–XY plane at intervals of 45 degrees, without any restrictions on the total range of angles [80]. A similar macrophage-based microrobot concept was developed by Bai et al. Specifically, γFe_2_O_3_ nanoparticles were engulfed by the macrophages, allowing them to move toward the capillary wall through the radiation force induced via a PZT (lead zirconate titanate) transducer in a microfluidic channel. The microrobots could also be manipulated using an alternative magnetic field [81]. The use of acoustic targeting offers several advantages, such as deep tissue penetration, strong actuation force, long operation time, safe actuation sources, and the possibility of generation of ultrasound images of the microrobots [82]. However, accompanying disadvantages include inflexibility in working environments, limited operation space, and complicated integrating instrumentation systems.

### 3.4. Magnetic Targeting

Magnetic targeting is a powerful and widely used targeting technique for precise control of microrobots in targeted tissues. This technique offers numerous advantages, including untethered maneuverability, lack of fuel requirement, and compatibility with other systems. To be manipulated using a magnetic field, microrobots should contain magnetic nanoparticles. When a magnetic microrobot is exposed to an external magnetic field, it experiences induced force and torque, which can be described as follows:

F = ν(M × ∇)B(6)

T = νM × B(7)

In these equations, ν and M denote the volume and magnetization of the microrobot, respectively; ∇ represents the gradient symbol; and B is the magnetic flux in the region of interest of the magnetic actuating system [83]. 

**Figure 5:**
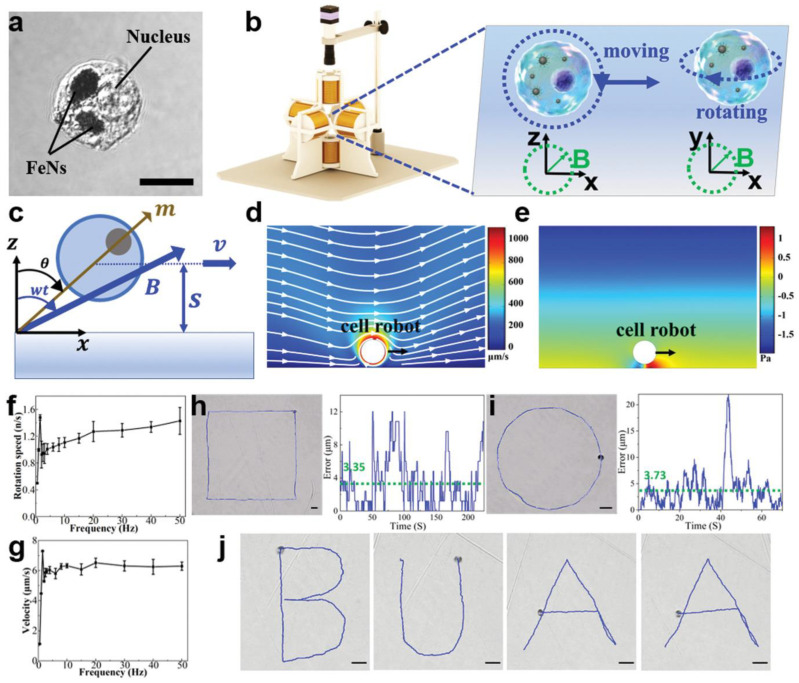
Precise control of the cell robot. (**a**) Confocal image of the cell robot, in which the black part indicates the internalized FeNs (scale bar: 10 µm). (**b**) Schematic of the magnetic generating system and two motion modes in the rotating magnetic field when near a wall. (**c**) Coordinate system of the rotating cell robot and the rotating magnetic field. (**d**) Simulation results of the flow streamlines on the XZ plane when the cell robot rotates and moves along the *x*-axis. (**e**) Simulation results of the pressure around the cell robot in the liquid environment near a wall. (**f**,**g**) Rotational velocity and moving velocity of the cell robot in the XZ plane versus magnetic field frequency. (**h**,**i**) Cell robot movement along a rectangular trajectory and a circle trajectory, respectively, and the corresponding tracking error (scale bar: 40 µm). (**j**) Trajectories of a cell robot in the rolling mode moving along a predefined “BUAA”-shaped track (scale bar: 40 µm). Reprinted with permission from ref. [86]. Copyright 2022, Wiley-VCH GmbH, Weinheim, Germany.

Two types of magnetic targeting commonly used for microrobot control are the electromagnet actuating (EMA) system and the permanent magnetic actuating (PMA) system. The EMA system consists of electromagnetic coils connected to power supplies that control the current of the coils through computer software. This system can generate a uniform magnetic field, magnetic gradient, and/or rotating magnetic fields to control the microrobots. The PMA system is composed of a set of permanent magnets, and the control of the microrobots is achieved by changing the distance and position of the magnets [84]. Each system has its own advantages and disadvantages. The EMA system allows for the generation of a magnetic field in a 3D space. In addition, the magnetic current and frequency can be easily adjusted, providing flexible and precise control. Dai et al. developed a magnetic manipulation platform (MMP) to control a magnetized macrophage-based microrobot carrying Dox-modified MNPs encapsulated liposomes. The MMP included a control computer, an AD/DA module, four digital drivers, and a magnetic field generator. This system could generate a magnetic field in any direction within the working space. Using this system, 80% of the cell microrobots could be controlled to target reservoirs in a microfluidic channel, and most of the cells remained alive at the targeted sites [85]. Sitti’s group used a five-coil EMA system, capable of generating a rotating magnetic field in the range of 1–20 mT, to manipulate macrophage-based microrobots (immunobots) carrying magnetic decoy bacteria Janus particles. The macrophages used were either J774A.1 or mouse bone marrow-derived macrophages (Figure 5). A 10 mT rotating magnetic field was applied under static and flow conditions. Under static conditions, the maximum average velocity reached 82.2 µm/s at a step-out frequency of 30 Hz. Under a flow condition of 2 mm/s, the microrobots could be reliably steered against the flow with an applied 10 mT and 30 Hz magnetic field. However, at a flow rate of 4 mm/s, the fluidic drag balanced with the magnetic force, resulting in no net displacement [29]. Han et al. adopted an EMA system that included three pairs of Helmholtz coils to generate uniform magnetic flux and two pairs of Maxwell coils to create a uniform magnetic gradient in the area of interest. Under a magnetic gradient of 10 mT/m, the macrophage-based microrobots could be manipulated to reach a velocity of 63 µm/s [55]. The MΦ-OMVs robots developed by Li et al. could be controlled within a wide range of magnetic field intensities (5.1–18 mT) and frequencies (8–24 Hz), with an average velocity of 25.9 μm/s achieved at an intensity of 16.1 mT and a 16 Hz frequency [48]. The macrophage-based microrobots (cell robots) prepared by Dai et al. using PLL@Fe_2_O_3_ (FeNs) nanoparticles and macrophages could be controlled with increased velocity at a low frequency and kept almost constant at a velocity of 6 μm/s after the step-out frequency (Figure 5) [86]. 

However, the EMA system has its drawbacks, including cost, space requirements, and heat generation. Therefore, in many studies, PMA systems are employed to manipulate the macrophage microrobots. Compared to the EMA system, the PMA system does not require complex coils, control interfaces, or power supplies, eliminating the overheating problem. In addition, the induced magnetic flux density can be much higher. However, the control flexibility of the magnetic field is limited, and it is difficult to cease the magnetic field during operation [84]. In terms of using a PMA system to manipulate macrophage-based microrobots, Fujita et al. used a magnet to control macrophages magnetized with magnetic lipoplexes, showing that under a magnetic field, the macrophages remarkably adhered to the murine colon in vivo [46]. Recently, our team developed a macrophage-based microrobot using primary macrophages isolated from a murine spleen. The microrobot carried both citric acid-coated MNPs and thermosensitive Dox liposomes. Using a permanent magnet, the targeting and therapeutic effect of the macrophage microrobots in 4T1 breast cancer tumor-bearing mice were significantly improved [87]. Besides the two aforementioned types of magnetic actuating systems, a commercialized magnetic resonance imaging (MRI) system could be used to target macrophage-based microrobots. Lewis and colleagues utilized pulsed magnetic field gradients to direct the macrophages that were magnetized with super-paramagnetic iron oxide nanoparticles (SPIONs) carrying the oncolytic virus Seprehvir toward tumors. They showed that the macrophage infiltration into the tumor was increased and the tumor burden and metastasis were reduced [88].

## 4. Applications of Macrophage-Based Microrobots for Anticancer Therapy

### 4.1. Photothermal Therapy

**Figure 6:**
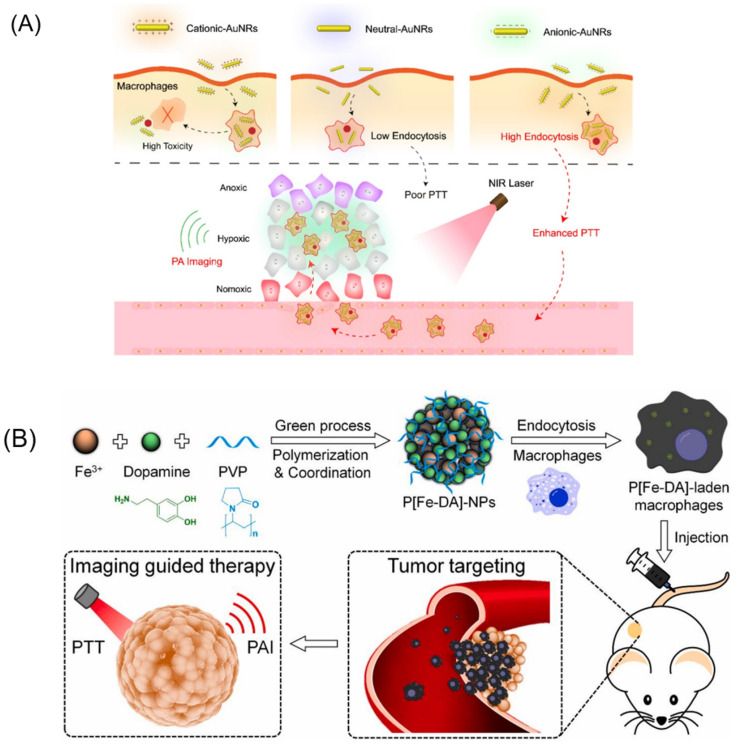
(**A**) Design and application of AuNR-laden macrophages for tumor hypoxia-targeted PA imaging and enhanced PTT. Reprinted with permission from ref. [24]. Copyright 2019, American Chemical Society. (**B**) Schematic illustration of the preparation of P[Fe-DA]-NPs and in vivo theranostic application of P[Fe-DA]-laden macrophages. P[Fe-DA]-NPs were successfully prepared using Fe^3+^, DA, and PVP based on a one-step method and were endocytosed via macrophages to construct the P[Fe-DA]-laden macrophage delivery systems. Then, P[Fe-DA]-laden macrophages were injected into 4T1 tumor-bearing BALB/c mice via the tail vein, and targeted the tumors. Effective PTT could be achieved under 808 nm light irradiation with PAI used as a guide. Reprinted with permission from ref. [57]. Copyright 2021, Elsevier.

Photothermal therapy (PTT) is a method used to treat cancer by converting light energy into heat energy, which then induces local hyperthermia to destroy cancer cells [89]. In PTT, NIR light is typically used due to its ability to penetrate deep into tissues [90]. Generally, once NIR light-sensitive agents are targeted toward cancer sites, they are irradiated with an NIR laser to increase the temperature at these sites to a level sufficient to kill cancer cells. In a study by Baek et al., gold nanoshells were loaded into macrophages to investigate their potential for treating gliomas. The researchers found that the nanoshell-loaded macrophages could completely inhibit tumor growth in an NIR irradiance-dependent manner [91]. Similarly, Madsen et al. used PEGylated gold nanoshells loaded into rat alveolar macrophages to treat a glioma and showed that the macrophages loaded with nanoshells could prevent or delay the tumor’s development [31,92]. Small gold nanorods are also widely used with macrophages and have promising therapeutic outcomes (Figure 6A) [24,25,45]. Recently, Zhang et al. have reported the use of poly-(iron-dopamine coordination complex) nanoparticles (P[Fe-DA]-NPs), which exhibit both NIR light sensitivity and excellent photoacoustic imaging (PAI) properties, loaded into RAW 264.7 macrophages to generate microrobots (Figure 6B). These microrobots, when injected into 4T1 tumor-bearing nude mice, significantly suppressed tumor growth after PTT treatment (1.0 W cm^−2^ for 5 min) under the guidance of PAI [57]. 

### 4.2. Chemotherapy

**Figure 7:**
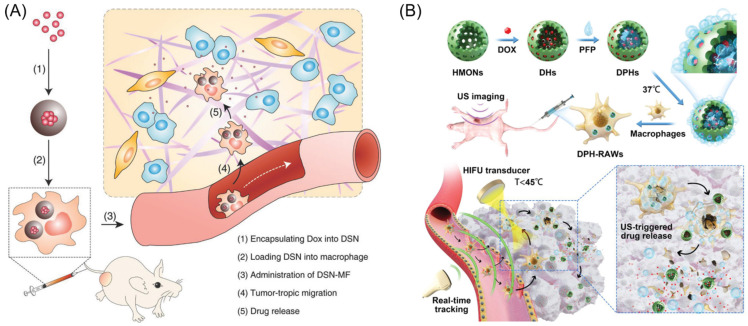
(**A**) Nanocapsule-laden macrophages for drug delivery to tumors. (1) An antineoplastic drug, in this particular case Dox, was first loaded into a carefully tailored nanocapsule called drug–silica nanocomplex (DSN). (2) DSN nanoparticles were engulfed by macrophages ex vivo. (3) DSN-laden macrophages (DSN-MF) were intravenously injected into a tumor-bearing mouse. (4) Chemotactic migration of DSN-MF to tumors. (5) DSN-MF releases Dox inside the tumor to selectively kill cancer cells. Reprinted with permission from ref. [94]. Copyright 2018, WILEY-VCH Verlag GmbH & Co. KGaA, Weinheim. (**B**) Schematic design of macrophage-mediated drug delivery system. DOX and PFP were loaded into the HMONs to form DPHs, which were then incubated with macrophages at 37 °C. A small portion of the PFP generated small bubbles that enabled real-time ultrasound tracking. DPH-loaded macrophages (DPH-RAWs) exhibited enhanced tumor accumulation, extravasation from tumor vessels, and penetration into the deeper layers of the tumor tissue. After short-pulsed HIFU sonication, the remaining PFP vaporized to form numerous large bubbles that ruptured the macrophages, resulting in DOX release and excellent anticancer activity. Reprinted with permission from ref. [95]. Copyright 2020, WILEY-VCH Verlag GmbH & Co. KGaA, Weinheim, Germany.

Chemotherapy for cancer involves the use of potent drugs to eliminate cancer cells. However, these drugs often cause side effects when administered to patients. Macrophage-based microrobots that possess the ability to target tumors could potentially reduce side effects by delivering drugs directly to specific sites. In addition, the controlled release of drugs from the macrophages could enhance the therapeutic effects of the drugs. In a study by Weissleder et al., nanotherapeutics consisting of fluorescent platinum (IV) prodrugs and a clinically tested polymer platform (PLGA-b-PEG) were slowly released from tumor-associated macrophages when infused into mice [93]. Xie et al. reported a successful loading of DOX into macrophages (16.6 pg per cell) using silica nanocapsules (Figure 7A). When injected intravenously into a U87MG xenograft model, these nanocapsule-laden macrophages efficiently suppressed tumor growth while causing minimal systemic toxicity [94]. Recently, Xu et al. developed macrophage-based microrobots by incorporating DOX and perfluoropentane into hollow mesoporous organosilica nanoparticles and allowing them to become engulfed into RAW 264.7 macrophages (Figure 7B). Once targeted toward tumors in vivo, the microrobots were treated with high-intensity focused ultrasound in order to generate microbubbles to kill the macrophages and release the DOX, significantly inhibiting tumor growth [95].

Godin and colleagues loaded nanoporous multistage vectors encapsulated with nanoparticle albumin-bound paclitaxel into macrophages. In vivo experiments showed that this system significantly increased the number of macrophages in the liver compared with untreated groups, suggesting its potential for treating metastatic liver cancers [96].

### 4.3. Immunotherapy

**Figure 8:**
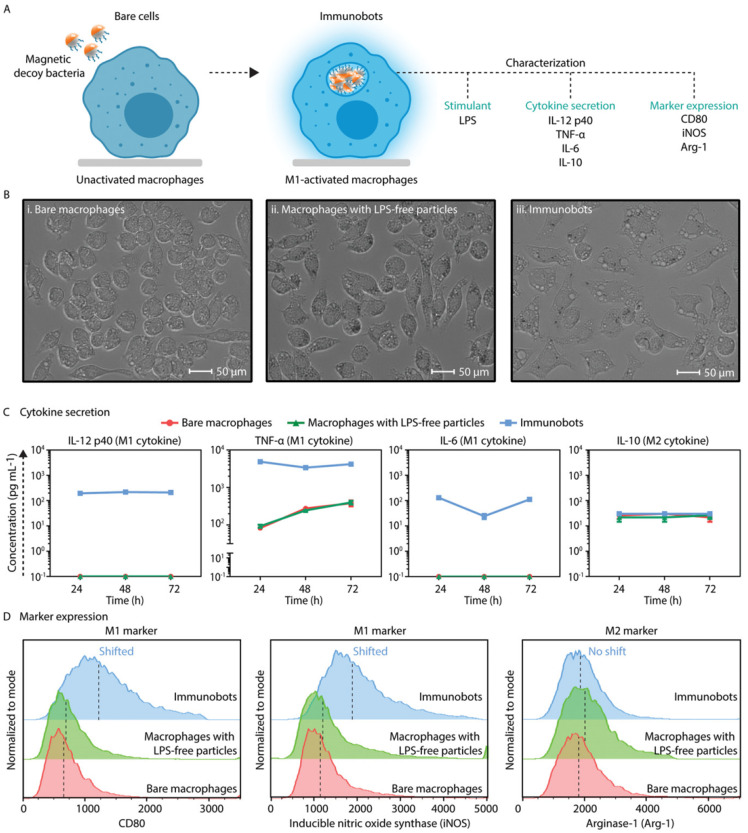
Regulating and sustaining phenotypes of macrophages toward an anti-tumorigenic state. (**A**) A summary of modulating the phenotypes of macrophages. The stimulation of macrophages with bacterial LPS units of magnetic decoy bacteria activated the release of specific cytokines and enhanced the expression levels of cell markers. (**B**) Optical microscope DIC images of (i) bare macrophages, (ii) macrophages treated with LPS-free particles, and (iii) immunobots. Incubation with magnetic decoy bacteria overnight increased cell size and resulted in distinct morphological changes. (**C**) Analysis of the macrophages’ phenotypic states was performed by measuring the levels of M1 cytokines (IL-12 p40, TNF-α, and IL-6) and M2 cytokines (IL-10). The immunobot group produced higher amounts of M1 cytokines compared to bare macrophages and macrophages treated with LPS-free particles, whereas the M2 cytokine was secreted at the same level for all groups. (**D**) The expressions of M1 markers (CD80 and iNOS) and M2 marker (Arg-1) on macrophages were determined via flow cytometry (n = 10,000 events per data point). The presence of bacterial LPS in the case of the immunobots triggered a higher expression of M1 markers without showing any significant change in M2 marker expression. Reprinted from ref. [29] under the terms of the CC-BY license.

When appropriately activated, macrophages can destroy tumor cells through phagocytosis and interact with components of the immune system [97,98]. Macrophages can be activated into two different phenotypes: M2, which is a tumor-associated macrophage that promotes tumor growth, and M1, a proinflammatory macrophage that digests tumor cells [99]. Therefore, to enable the immunotherapy of cancers using macrophage-based microrobots, critical efforts have been made to ensure that the payloads of the microrobots could induce the activation of the carrying macrophages toward an M1 phenotype. Recently, Kumari et al. prepared self-assembled amphiphilic PEGylated galactomannan nanoparticles loaded with hydrazinocurcumin, which has the ability to re-regulate M2 to M1 macrophages, and treated them with IL-4 -activated RAW 264.7 macrophages (M2). In vivo experiments in EAC-bearing mice showed that the nanoparticles reduced tumor burden and increased survival rate [100]. The immunobots created by Sitti et al. were constructed using magnetic decoy bacteria-coated silica Janus microbeads [29]. The bacterial lipopolysaccharide could induce macrophages toward anti-tumorigenic (M1) phenotypes. Experiments showed that the microrobots released important M1 cytokines, including IL-12 p40, TNF-α, and IL-6, as well as M1 phenotype markers including CD80 and iNOS bacterial lipopolysaccharide activation (Figure 8). In addition, the microrobots displayed anticancer abilities when tested with urinary bladder cancer cells.

### 4.4. Combination Therapy

**Figure 9:**
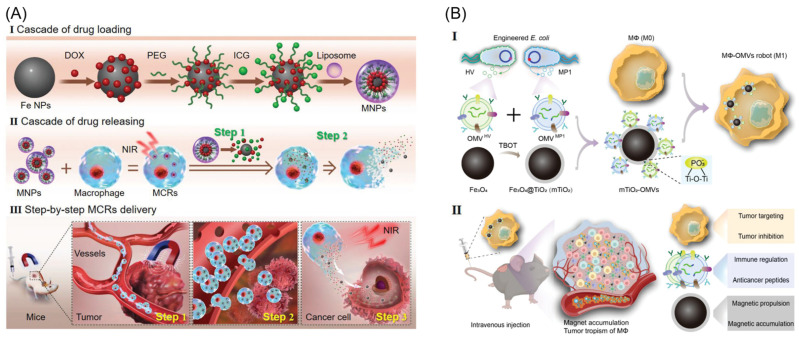
(**A**) Schematic of the magnetic control system, and illustration of cell robots in operation. (i) Schematic of the fabrication of the MNPs. (ii) Fabrication of the cell robot, and the release of DOX from MNPs and MCRs. (iii) Summary of in vivo tumor targeting and therapy with the cell robot. Reprinted with permission from ref. [85]. Copyright 2021, Wiley-VCH GmbH. (**B**) Schematic of the fabrication of MΦ-OMV robots and their targeted multimodal therapy against the subcutaneous tumor. (i) Bacteria were genetically engineered to express anticancer peptides, HV and MP1, yielding two types of OMVs, OMVHV, and OMVMP1. TiO_2_-coated Fe_3_O_4_ NPs were used to capture such two OMVs, which were then endocytosed by macrophages to create MΦ-OMVs robots. (ii) Magnetic manipulation and tumor tropism of the macrophage body can synergistically enhance the accumulation of intravenously injected cell robots at the subcutaneous tumor site, further improving the antitumor efficacy of cell robots capable of multiple treatment modes, including tumor suppression of the macrophage body, anticancer immunity modulation, and antitumor peptides of OMVs. Reprinted with permission from ref. [48]. Copyright 2023, Wiley-VCH GmbH.

To enhance the therapeutic outcomes of macrophage-based microrobots, researchers have been attempting to combine different monotherapies in a single cellular platform [56]. Photothermal–chemo combination therapy has been commonly adopted [101]. The microrobots designed and fabricated by Dai et al., with MNPs functionalized with DOX and indocyanine (ICG), allowed both chemotherapy using DOX and PTT via ICG under NIR irradiation (Figure 9A). As a result, effective and targeted therapy using the microrobots in tumor-bearing mice was demonstrated [85]. Our group recently constructed a macrophage-based microrobot using RAW 264.7 macrophages internalized with small-sized gold nanorods and DOX-loaded liposomes. Upon targeting solid tumors in vivo, the microrobots were irradiated with an NIR laser that increased the local temperature of the tumors and triggered DOX release from the liposomes. Consequently, using PTT with chemotherapy, the growth of the solid tumors was significantly inhibited by these microrobots [102]. Fang et al. recently fabricated a macrophage-based microrobot by adsorbing DOX-loaded poly-(lactic-co-glycolic acid) nanoparticles onto a macrophage cell surface mediated with a divalent metal ion-phenolic network. Thus, compared to internalization of the nanoparticles into the macrophages, attaching them to the cell surface would reduce drug toxicity and facilitate enhanced drug release at the tumor sites. As a result, the treatment with microrobots and laser irradiation showed the strongest antitumor effect in an orthotopic 4T1 breast cancer model. In addition, lung metastasis of 4T1 tumors was also significantly reduced after the treatment [103]. Macrophage-based microrobots could also be used for image-guided combination therapy that allows the targeted and precise killing of cancer cells. For this purpose, Zhang et al. conjugated IR-820 to macrophages for image-guided functions and loaded into them pH-sensitive DOX nanoparticles, enabling PTT via NIR irradiation and chemotherapy via the released DOX. In vivo experiments showed an excellent therapeutic effect against 4T1 tumors in Balb/C mice using three injections of the microrobots and five doses of NIR irradiation [104]. The combination of chemotherapy, photodynamic therapy, and immunotherapy using macrophage-based microrobots was also explored [26]. Using this strategy, Huang et al. isolated primary macrophages from C57BL/6 mice’s bone marrow and loaded them with oxaliplatin prodrug and photosensitizer-carrying nanoparticles (Oxa(IV)@ZnPc@M). The microrobots showed an M1 tumoricidal phenotype and an ability to target primary and metastatic bone tumors in vivo. In addition, they could effectively kill tumor cells using chemo-photodynamic therapy in combination with immunogenic cell death via co-administration with anti-PD-L1 [105]. Hou et al. used M1 macrophages to ferry sorafenib-loaded lipid nanoparticles (M1/SLNP). These researchers showed an enhanced portion of M1 macrophages, compared to M2-type macrophages, and quantities of the CD3+ CD4+ T cells and CD3+ CD8+ T cells in tumors after treatment with M1/SLNP, enabling effective cell–chemotherapy [106]. Wang et al. proposed the use of macrophages for synergistic chemo-gene-immunotherapy by loading the cells with functional nucleic acid therapeutics and the chemotherapeutic drug cisplatin. It was reported that after loading them with therapeutics, the macrophages could carry the payloads toward the tumor sites and activate and retain their immunostimulatory effects [107]. A multimodal anticancer therapy using the macrophage-based microrobots (MΦ-OMVs robots) actively targeted the tumors using magnetic guidance as proposed by Li et al. [48]. The MΦ-OMVs robots with payloads containing bioengineered bacterial outer-membrane vesicles and magnetic nanoparticles could actively target tumors using magnetic fields, promoting tumor inhibition using macrophages and peptides, with good biocompatibility and minor side effects (Figure 9B).

Detailed applications of the macrophage-based microrobots for anticancer therapy are summarized in Table 1.

## 5. Challenges and Future Perspectives

**Figure 10:**
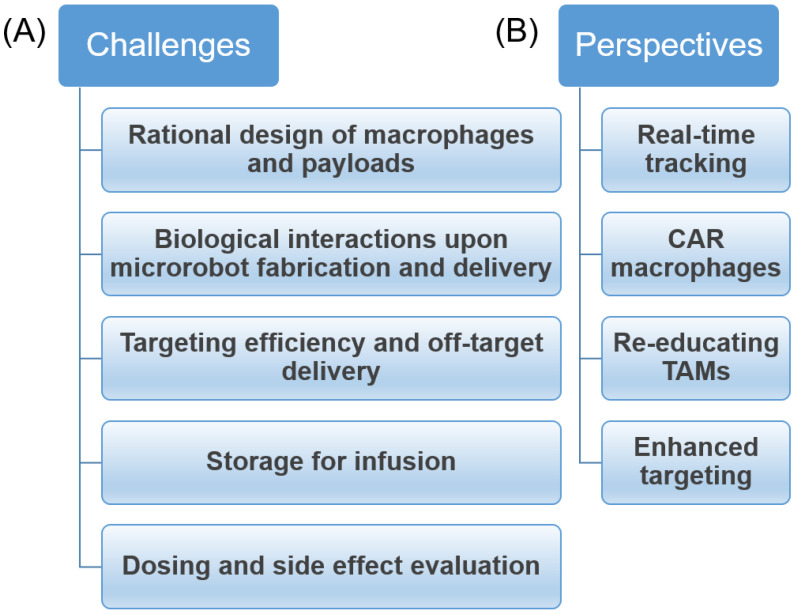
Challenges and future perspectives for the development of macrophage-based microrobots for anticancer therapy. (**A**) Challenges; (**B**) Perspectives.

Although great efforts have been made to develop an effective anticancer therapy using macrophages with promising therapeutic outcomes both in vitro and in vivo, this field is in its immature stage and many challenges exist (Figure 10A). First, the rational design of the combination of macrophages and payloads has yet to be well studied [108]. Second, insights of the biological interactions between them for microrobot fabrication and anticancer therapy should be well investigated. Third, the targeting efficiency, including the off-target delivery of microrobots, has yet to be well examined, including the survival rate of the microrobots at the targeted sites [109]. Fourth, the appropriate method of storing the microrobots after fabrication to maintain their viability and functionality is also a challenge and should be highly considered [110]. Finally, while the benefit of using macrophage-based microrobots has been shown to be promising in anticancer therapy from the reviewed data above, the dosing of the microrobots and the side effects upon their infusion into the body should be critically investigated in order to understand the risks of using this new form of delivery system. Therefore, there are several aspects that could be explored in future research (Figure 10B). First of all, the targeting or control of the microrobots to the tumors should be visualized with a real-time tracking function using advanced imaging techniques that allow precise targeting and releasing of payloads. In addition, chimeric antigen receptor (CAR) macrophages could be used to construct the microrobots, which would allow for effective treatment of cancer utilizing immunotherapy. Furthermore, re-educating macrophages from tumor-associated macrophages (TAMs) to tumoricidal macrophages is also considered an important strategy in creating anticancer macrophage-based microrobots. However, changes in the phenotypes of the macrophages may not be stable. Consequently, if macrophages are regulated to M1 macrophages with some stimulations ex vivo, when injected back into the host body, the M1 phenotype may not be maintained. Accordingly, it could be better if TAMs are repolarized to M1 at the tumor site, as the therapeutic outcomes would be improved [111,112,113,114,115]. Moreover, to enhance the targeting ability of macrophages, they could be embedded into other micro-sized biodegradable structures that allow for easier control via external actuating systems than the small-sized macrophage-based microrobots [116,117]. 

## 6. Conclusions

In this work, we reviewed the recent progress of using macrophage-based microrobots as a promising approach for anticancer therapy. We explained the different types of macrophage cells that have been commonly used in the fabrication of these microrobots, the popular payloads that have been used for carrying therapeutic agents of the microrobots, and various targeting methods that have been adopted to control and direct the microrobots toward cancer areas, including biological, chemical, acoustic, and magnetic actuation. Then, the applications of these microrobots in different cancer treatment therapies, including PTT, chemotherapy, immunotherapy, and combination therapies, were analyzed. Finally, we discussed the current challenges within this field and its future directions, including the risks and benefits associated with macrophage-based microrobots used for anticancer therapy.

## Figures and Tables

**Table 1:** Macrophage-based microrobots for anticancer therapy.

No.	Macrophage Type	Payload	Targeting Method	Therapy	Ref.
1	Ma P388-D1	Gold nanoshells	Biological	PTT	[91]
2	RAW 264.7	P[Fe-DA]-NPs	Biological	PTT	[57]
3	RAW 264.7	PLGA-b-PEG drug–silica nanocomplex (DSN)	Biological	Chemotherapy	[94]
4	RAW 264.7	DOX/PFP-loaded HMONs	Biological	Chemotherapy of breast cancer	[95]
5	J774A.1 and primary bone marrow macrophages	Magnetic decoy bacteria	Magnetic	Immunotherapy	[29]
6	RAW 264.7	Liposomes containing: MNPs, DOX, ICG	Magnetic	PTT–chemotherapy combination	[85]
7	Primary macrophages from a murine spleen	MNPs, DOX-Lip	Biological, Magnetic	PTT–chemotherapy combination	[87]
8	RAW 264.7	AuNRs, DOX-Lip	Biological	PTT–chemotherapy combination	[102]
9	RAW 264.7	DOX-MPN	Biological	PTT–chemotherapy combination	[103]
10	RAW 264.7	IR-RC, DOX-NPs	Biological	PTT–chemotherapy combination	[104]
11	RAW 264.7	MDM	Biological	Photodynamic–chemotherapy combination	[26]
12	Primary macrophages	Oxa(IV)@ZnPc@M	Biological	Photodynamic–chemotherapy combination	[105]
13	RAW 264.7	SLNP	Biological	Cell–chemotherapy combination	[106]
14	RAW 264.7	CpG-ASO-Pt	Biological	Chemo-gene-immunotherapy	[107]
15	RAW 264.7	mTiO_2_-OMVs	Magnetic, Biological	Multimodal therapy	[48]
16	RAW 264.7	PLL@Mg/TiO_2_	Chemical		[67]
17	RAW 264.7	SPIONs	Acoustic		[80]
18	J774A.1	PLGA-DTX-Fe_3_O_4_	Magnetic, Biological	Chemotherapy	[55]
19	RAW 264.7	AuNRs	Biological	PTT	[45]
20	RAW 264.7	Free DOX	Biological	Chemotherapy	[58]

## Data Availability

Not applicable.

## References

[1] Lee H., Kim D.-i., Kwon S.-h., Park S. (2021). Magnetically Actuated Drug Delivery Helical Microrobot with Magnetic Nanoparticle Retrieval Ability. ACS Appl. Mater. Interfaces.

[2] Ceylan H., Yasa I.C., Yasa O., Tabak A.F., Giltinan J., Sitti M. (2019). 3D-Printed Biodegradable Microswimmer for Theranostic Cargo Delivery and Release. ACS Nano.

[3] Dong M., Wang X., Chen X.-Z., Mushtaq F., Deng S., Zhu C., Torlakcik H., Terzopoulou A., Qin X.-H., Xiao X. (2020). 3D-Printed Soft Magnetoelectric Microswimmers for Delivery and Differentiation of Neuron-like Cells. Adv. Funct. Mater..

[4] Ceylan H., Dogan N.O., Yasa I.C., Musaoglu M.N., Kulali Z.U., Sitti M. (2021). 3D printed personalized magnetic micromachines from patient blood derived biomaterials. Sci. Adv..

[5] Adam G., Benouhiba A., Rabenorosoa K., Clévy C., Cappelleri D.J. (2021). 4D Printing: Enabling Technology for Microrobotics Applications. Adv. Intell. Syst..

[6] Ye J., Wilson D.A., Tu Y., Peng F. (2020). 3D-Printed Micromotors for Biomedical Applications. Adv. Mater. Technol..

[7] Gong D., Celi N., Zhang D., Cai J. (2022). Magnetic Biohybrid Microrobot Multimers Based on Chlorella Cells for Enhanced Targeted Drug Delivery. ACS Appl. Mater. Interfaces.

[8] Liu L., Wu J., Chen B., Gao J., Li T., Ye Y., Tian H., Wang S., Wang F., Jiang J. (2022). Magnetically Actuated Biohybrid Microswimmers for Precise Photothermal Muscle Contraction. ACS Nano.

[9] Gong D., Sun L., Li X., Zhang W., Zhang D., Cai J. (2023). Micro/Nanofabrication, Assembly, and Actuation Based on Microorganisms: Recent Advances and Perspectives. Small Struct..

[10] Zhang F., Zhuang J., Li Z., Gong H., de Ávila B.E.-F., Duan Y., Zhang Q., Zhou J., Yin L., Karshalev E. (2022). Nanoparticle-modified microrobots for in vivo antibiotic delivery to treat acute bacterial pneumonia. Nat. Mater..

[11] Chen W., Wang Y., Qin M., Zhang X., Zhang Z., Sun X., Gu Z. (2018). Bacteria-Driven Hypoxia Targeting for Combined Biotherapy and Photothermal Therapy. ACS Nano.

[12] Park B.-W., Zhuang J., Yasa O., Sitti M. (2017). Multifunctional Bacteria-Driven Microswimmers for Targeted Active Drug Delivery. ACS Nano.

[13] Fan J.-X., Li Z.-H., Liu X.-H., Zheng D.-W., Chen Y., Zhang X.-Z. (2018). Bacteria-Mediated Tumor Therapy Utilizing Photothermally-Controlled TNF-α Expression via Oral Administration. Nano Lett..

[14] Luo C.-H., Huang C.-T., Su C.-H., Yeh C.-S. (2016). Bacteria-Mediated Hypoxia-Specific Delivery of Nanoparticles for Tumors Imaging and Therapy. Nano Lett..

[15] Felfoul O., Mohammadi M., Taherkhani S., de Lanauze D., Zhong Xu Y., Loghin D., Essa S., Jancik S., Houle D., Lafleur M. (2016). Magneto-aerotactic bacteria deliver drug-containing nanoliposomes to tumour hypoxic regions. Nat. Nanotechnol..

[16] Zheng D.-W., Chen Y., Li Z.-H., Xu L., Li C.-X., Li B., Fan J.-X., Cheng S.-X., Zhang X.-Z. (2018). Optically-controlled bacterial metabolite for cancer therapy. Nat. Commun..

[17] Xin C., Jin D., Hu Y., Yang L., Li R., Wang L., Ren Z., Wang D., Ji S., Hu K. (2021). Environmentally Adaptive Shape-Morphing Microrobots for Localized Cancer Cell Treatment. ACS Nano.

[18] Contreras-Llano L.E., Liu Y.-H., Henson T., Meyer C.C., Baghdasaryan O., Khan S., Lin C.-L., Wang A., Hu C.-M.J., Tan C. (2023). Engineering Cyborg Bacteria Through Intracellular Hydrogelation. Adv. Sci..

[19] Baghdasaryan O., Khan S., Lin J.-C., Lee-Kin J., Hsu C.-Y., Hu C.-M.J., Tan C. (2023). Synthetic control of living cells by intracellular polymerization. Trends Biotechnol..

[20] Wang H.F., Liu Y., Yang G., Zhao C.X. (2021). Macrophage-mediated cancer drug delivery. Mater. Today Sustain..

[21] Lendeckel U., Venz S., Wolke C. (2022). Macrophages: Shapes and functions. ChemTexts.

[22] Gui Y., Zeng Y., Chen B., Yang Y., Ma J., Li C. (2023). A smart pathogen detector engineered from intracellular hydrogelation of DNA-decorated macrophages. Nat. Commun..

[23] Wang J., Hu D., Chen Q., Liu T., Zhou X., Xu Y., Zhou H., Gu D., Gao C. (2023). Intracellular hydrogelation of macrophage conjugated probiotics for hitchhiking delivery and combined treatment of colitis. Mater. Today Bio.

[24] An L., Wang Y., Lin J., Tian Q., Xie Y., Hu J., Yang S. (2019). Macrophages-Mediated Delivery of Small Gold Nanorods for Tumor Hypoxia Photoacoustic Imaging and Enhanced Photothermal Therapy. ACS Appl. Mater. Interfaces.

[25] Oh N., Kim Y., Kweon H.-S., Oh W.-Y., Park J.-H. (2018). Macrophage-Mediated Exocytosis of Elongated Nanoparticles Improves Hepatic Excretion and Cancer Phototherapy. ACS Appl. Mater. Interfaces.

[26] Sun P., Deng Q., Kang L., Sun Y., Ren J., Qu X. (2020). A Smart Nanoparticle-Laden and Remote-Controlled Self-Destructive Macrophage for Enhanced Chemo/Chemodynamic Synergistic Therapy. ACS Nano.

[27] Min J.H., Kim S.T., Lee J.S., Kim K., Wu J.H., Jeong J., Song A.Y., Lee K.-M., Kim Y.K. (2011). Labeling of macrophage cell using biocompatible magnetic nanoparticles. J. Appl. Phys..

[28] Guo L., Zhang Y., Yang Z., Peng H., Wei R., Wang C., Feng M. (2019). Tunneling Nanotubular Expressways for Ultrafast and Accurate M1 Macrophage Delivery of Anticancer Drugs to Metastatic Ovarian Carcinoma. ACS Nano.

[29] Dogan N.O., Ceylan H., Suadiye E., Sheehan D., Aydin A., Yasa I.C., Wild A.-M., Richter G., Sitti M. (2022). Remotely Guided Immunobots Engaged in Anti-Tumorigenic Phenotypes for Targeted Cancer Immunotherapy. Small.

[30] Du Nguyen V., Le V.H., Zheng S., Han J., Park J.-O. (2018). Preparation of tumor targeting cell-based microrobots carrying NIR light sensitive therapeutics manipulated by electromagnetic actuating system and Chemotaxis. J. Micro-Bio Robot..

[31] Madsen S.J., Christie C., Hong S.J., Trinidad A., Peng Q., Uzal F.A., Hirschberg H. (2015). Nanoparticle-loaded macrophage-mediated photothermal therapy: Potential for glioma treatment. Lasers Med. Sci..

[32] Xie Z., Su Y., Kim G.B., Selvi E., Ma C., Aragon-Sanabria V., Hsieh J.-T., Dong C., Yang J. (2017). Immune Cell-Mediated Biodegradable Theranostic Nanoparticles for Melanoma Targeting and Drug Delivery. Small.

[33] Park S.J., Lee Y., Choi Y.J., Cho S., Jung H.E., Zheng S., Park B.J., Ko S.Y., Park J.O., Park S. (2014). Monocyte-based microrobot with chemotactic motility for tumor theragnosis. Biotechnol. Bioeng..

[34] Choi J., Kim H.Y., Ju E.J., Jung J., Park J., Chung H.K., Lee J.S., Lee J.S., Park H.J., Song S.Y. (2012). Use of macrophages to deliver therapeutic and imaging contrast agents to tumors. Biomaterials.

[35] Pang L., Zhu Y., Qin J., Zhao W., Wang J. (2018). Primary M1 macrophages as multifunctional carrier combined with PLGA nanoparticle delivering anticancer drug for efficient glioma therapy. Drug Deliv..

[36] Cao H., Wang H., He X., Tan T., Hu H., Wang Z., Wang J., Li J., Zhang Z., Li Y. (2018). Bioengineered Macrophages Can Responsively Transform into Nanovesicles To Target Lung Metastasis. Nano Lett..

[37] Dou H., Destache C.J., Morehead J.R., Mosley R.L., Boska M.D., Kingsley J., Gorantla S., Poluektova L., Nelson J.A., Chaubal M. (2006). Development of a macrophage-based nanoparticle platform for antiretroviral drug delivery. Blood.

[38] Evans M.A., Huang P.-J., Iwamoto Y., Ibsen K.N., Chan E.M., Hitomi Y., Ford P.C., Mitragotri S. (2018). Macrophage-mediated delivery of light activated nitric oxide prodrugs with spatial, temporal and concentration control. Chem. Sci..

[39] Choi M.-R., Stanton-Maxey K.J., Stanley J.K., Levin C.S., Bardhan R., Akin D., Badve S., Sturgis J., Robinson J.P., Bashir R. (2007). A Cellular Trojan Horse for Delivery of Therapeutic Nanoparticles into Tumors. Nano Lett..

[40] Yu L., Zhu S., Qin K., Fan X., An L. (2022). Macrophages Loaded with Fe Nanoparticles for Enhanced Photothermal Ablation of Tumors. J. Funct. Biomater..

[41] Doshi N., Swiston A.J., Gilbert J.B., Alcaraz M.L., Cohen R.E., Rubner M.F., Mitragotri S. (2011). Cell-Based Drug Delivery Devices Using Phagocytosis-Resistant Backpacks. Adv. Mater..

[42] Anselmo A.C., Gilbert J.B., Kumar S., Gupta V., Cohen R.E., Rubner M.F., Mitragotri S. (2015). Monocyte-mediated delivery of polymeric backpacks to inflamed tissues: A generalized strategy to deliver drugs to treat inflammation. J. Control. Release.

[43] Yang L., Zhang Y., Zhang Y., Xu Y., Li Y., Xie Z., Wang H., Lin Y., Lin Q., Gong T. (2022). Live Macrophage-Delivered Doxorubicin-Loaded Liposomes Effectively Treat Triple-Negative Breast Cancer. ACS Nano.

[44] Chen J., Lin L., Yan N., Hu Y., Fang H., Guo Z., Sun P., Tian H., Chen X. (2018). Macrophages loaded CpG and GNR-PEI for combination of tumor photothermal therapy and immunotherapy. Sci. China Mater..

[45] Li Z., Huang H., Tang S., Li Y., Yu X.-F., Wang H., Li P., Sun Z., Zhang H., Liu C. (2016). Small gold nanorods laden macrophages for enhanced tumor coverage in photothermal therapy. Biomaterials.

[46] Kono Y., Gogatsubo S., Ohba T., Fujita T. (2019). Enhanced macrophage delivery to the colon using magnetic lipoplexes with a magnetic field. Drug Deliv..

[47] Materón E.M., Miyazaki C.M., Carr O., Joshi N., Picciani P.H.S., Dalmaschio C.J., Davis F., Shimizu F.M. (2021). Magnetic nanoparticles in biomedical applications: A review. Appl. Surf. Sci. Adv..

[48] Li Y., Cong Z., Xie L., Tang S., Ren C., Peng X., Tang D., Wan F., Han H., Zhang X. (2023). Magnetically Powered Immunogenic Macrophage Microrobots for Targeted Multimodal Cancer Therapy. Small.

[49] Nguyen V.D., Han J., Go G., Zhen J., Zheng S., Le V.H., Park J.-O., Park S. (2017). Feasibility study of dual-targeting paclitaxel-loaded magnetic liposomes using electromagnetic actuation and macrophages. Sens. Actuators B Chem..

[50] Zhang L., Alimu G., Du Z., Yan T., Li H., Ma R., Lan Z., Yu Z., Alifu N., Sun K. (2023). Functionalized Magnetic Nanoparticles for NIR-Induced Photothermal Therapy of Potential Application in Cervical Cancer. ACS Omega.

[51] Zhang M., Cao Y., Wang L., Ma Y., Tu X., Zhang Z. (2015). Manganese Doped Iron Oxide Theranostic Nanoparticles for Combined T1 Magnetic Resonance Imaging and Photothermal Therapy. ACS Appl. Mater. Interfaces.

[52] Zheng R., Wang S., Tian Y., Jiang X., Fu D., Shen S., Yang W. (2015). Polydopamine-Coated Magnetic Composite Particles with an Enhanced Photothermal Effect. ACS Appl. Mater. Interfaces.

[53] Chu M., Shao Y., Peng J., Dai X., Li H., Wu Q., Shi D. (2013). Near-infrared laser light mediated cancer therapy by photothermal effect of Fe3O4 magnetic nanoparticles. Biomaterials.

[54] Shen S., Wang S., Zheng R., Zhu X., Jiang X., Fu D., Yang W. (2015). Magnetic nanoparticle clusters for photothermal therapy with near-infrared irradiation. Biomaterials.

[55] Han J., Zhen J., Nguyen V.D., Go G., Choi Y., Ko S.Y., Park J.-O., Park S. (2016). Hybrid-Actuating Macrophage-Based Microrobots for Active Cancer Therapy. Sci. Rep..

[56] Xiao T., Hu W., Fan Y., Shen M., Shi X. (2021). Macrophage-mediated tumor homing of hyaluronic acid nanogels loaded with polypyrrole and anticancer drug for targeted combinational photothermo-chemotherapy. Theranostics.

[57] Zhang X., Si Z., Wang Y., Li Y., Xu C., Tian H. (2021). Polymerization and coordination synergistically constructed photothermal agents for macrophages-mediated tumor targeting diagnosis and therapy. Biomaterials.

[58] Fu J., Wang D., Mei D., Zhang H., Wang Z., He B., Dai W., Zhang H., Wang X., Zhang Q. (2015). Macrophage mediated biomimetic delivery system for the treatment of lung metastasis of breast cancer. J. Control. Release.

[59] Aminin D., Wang Y.-M. (2021). Macrophages as a “weapon” in anticancer cellular immunotherapy. Kaohsiung J. Med. Sci..

[60] Ren K., Qiu Y., Yu Q., He J., Mei L., Liu Y., Li J., Wang X., Li M., Zhang Z. (2021). Macrophage-mediated multi-mode drug release system for photothermal combined with anti-inflammatory therapy against postoperative recurrence of triple negative breast cancer. Int. J. Pharm..

[61] Xiao Y., Zhang J., Fang B., Zhao X., Hao N. (2022). Acoustics-Actuated Microrobots. Micromachines.

[62] Villa K., Viktorova J., Plutnar J., Ruml T., Hoang L., Pumera M. (2020). Chemical Microrobots as Self-Propelled Microbrushes against Dental Biofilm. Cell Rep. Phys. Sci..

[63] Li J., Thamphiwatana S., Liu W., Esteban-Fernández de Ávila B., Angsantikul P., Sandraz E., Wang J., Xu T., Soto F., Ramez V. (2016). Enteric Micromotor Can Selectively Position and Spontaneously Propel in the Gastrointestinal Tract. ACS Nano.

[64] de Ávila B.E.-F., Angsantikul P., Li J., Angel Lopez-Ramirez M., Ramírez-Herrera D.E., Thamphiwatana S., Chen C., Delezuk J., Samakapiruk R., Ramez V. (2017). Micromotor-enabled active drug delivery for in vivo treatment of stomach infection. Nat. Commun..

[65] Li J., Angsantikul P., Liu W., Esteban-Fernández de Ávila B., Thamphiwatana S., Xu M., Sandraz E., Wang X., Delezuk J., Gao W. (2017). Micromotors Spontaneously Neutralize Gastric Acid for pH-Responsive Payload Release. Angew. Chem. Int. Ed..

[66] Gao W., Dong R., Thamphiwatana S., Li J., Gao W., Zhang L., Wang J. (2015). Artificial Micromotors in the Mouse’s Stomach: A Step toward in Vivo Use of Synthetic Motors. ACS Nano.

[67] Zhang F., Mundaca-Uribe R., Gong H., Esteban-Fernández de Ávila B., Beltrán-Gastélum M., Karshalev E., Nourhani A., Tong Y., Nguyen B., Gallot M. (2019). A Macrophage–Magnesium Hybrid Biomotor: Fabrication and Characterization. Adv. Mater..

[68] Zhang B., Pan H., Chen Z., Yin T., Zheng M., Cai L. (2023). Twin-bioengine self-adaptive micro/nanorobots using enzyme actuation and macrophage relay for gastrointestinal inflammation therapy. Sci Adv.

[69] Zhou H., Mayorga-Martinez C.C., Pané S., Zhang L., Pumera M. (2021). Magnetically Driven Micro and Nanorobots. Chem. Rev..

[70] Sánchez S., Soler L., Katuri J. (2015). Chemically Powered Micro- and Nanomotors. Angew. Chem. Int. Ed..

[71] Yang Y., Yang Y., Liu D., Wang Y., Lu M., Zhang Q., Huang J., Li Y., Ma T., Yan F. (2023). In-vivo programmable acoustic manipulation of genetically engineered bacteria. Nat. Commun..

[72] Wang J., Soto F., Ma P., Ahmed R., Yang H., Chen S., Wang J., Liu C., Akin D., Fu K. (2022). Acoustic Fabrication of Living Cardiomyocyte-based Hybrid Biorobots. ACS Nano.

[73] Wu Z., Li T., Li J., Gao W., Xu T., Christianson C., Gao W., Galarnyk M., He Q., Zhang L. (2014). Turning Erythrocytes into Functional Micromotors. ACS Nano.

[74] Ozcelik A., Rufo J., Guo F., Gu Y., Li P., Lata J., Huang T.J. (2018). Acoustic tweezers for the life sciences. Nat. Methods.

[75] Marzo A., Corkett T., Drinkwater B.W. (2018). Ultraino: An Open Phased-Array System for Narrowband Airborne Ultrasound Transmission. IEEE Trans. Ultrason. Ferroelectr. Freq. Control.

[76] Ghanem M.A., Maxwell A.D., Wang Y.-N., Cunitz B.W., Khokhlova V.A., Sapozhnikov O.A., Bailey M.R. (2020). Noninvasive acoustic manipulation of objects in a living body. Proc. Natl. Acad. Sci. USA.

[77] Franklin A., Marzo A., Malkin R., Drinkwater B.W. (2017). Three-dimensional ultrasonic trapping of micro-particles in water with a simple and compact two-element transducer. Appl. Phys. Lett..

[78] Yuan Z., Lu C., Liu C., Bai X., Zhao L., Feng S., Liu Y. (2023). Ultrasonic tweezer for multifunctional droplet manipulation. Sci. Adv..

[79] Marzo A., Seah S.A., Drinkwater B.W., Sahoo D.R., Long B., Subramanian S. (2015). Holographic acoustic elements for manipulation of levitated objects. Nat. Commun..

[80] Cao H.X., Nguyen V.D., Jung D., Choi E., Kim C.-S., Park J.-O., Kang B. (2022). Acoustically Driven Cell-Based Microrobots for Targeted Tumor Therapy. Pharmaceutics.

[81] Bai X., Zhang W., Dai Y., Wang Y., Sun H., Feng L. Acoustic and magnetic hybrid actuated immune cell robot for target and kill cancer cells. Proceedings of the 2022 International Conference on Robotics and Automation (ICRA).

[82] Huang L., Pan Y., Wang M., Ren L. (2023). Driving modes and characteristics of biomedical micro-robots. Eng. Regen..

[83] Wang M., Wu T., Liu R., Zhang Z., Liu J. (2023). Selective and Independent Control of Microrobots in a Magnetic Field: A Review. Engineering.

[84] Chen B., Sun H., Zhang J., Xu J., Song Z., Zhan G., Bai X., Feng L. (2023). Cell-Based Micro/Nano-Robots for Biomedical Applications: A Review. Small.

[85] Dai Y., Bai X., Jia L., Sun H., Feng Y., Wang L., Zhang C., Chen Y., Ji Y., Zhang D. (2021). Precise Control of Customized Macrophage Cell Robot for Targeted Therapy of Solid Tumors with Minimal Invasion. Small.

[86] Dai Y., Jia L., Wang L., Sun H., Ji Y., Wang C., Song L., Liang S., Chen D., Feng Y. (2022). Magnetically Actuated Cell-Robot System: Precise Control, Manipulation, and Multimode Conversion. Small.

[87] Nguyen V.D., Min H.-K., Kim H.Y., Han J., Choi Y.H., Kim C.-S., Park J.-O., Choi E. (2021). Primary Macrophage-Based Microrobots: An Effective Tumor Therapy In Vivo by Dual-Targeting Function and Near-Infrared-Triggered Drug Release. ACS Nano.

[88] Muthana M., Kennerley A.J., Hughes R., Fagnano E., Richardson J., Paul M., Murdoch C., Wright F., Payne C., Lythgoe M.F. (2015). Directing cell therapy to anatomic target sites in vivo with magnetic resonance targeting. Nat. Commun..

[89] Zhao L., Zhang X., Wang X., Guan X., Zhang W., Ma J. (2021). Recent advances in selective photothermal therapy of tumor. J. Nanobiotechnology.

[90] Chechetka S.A., Yuba E., Kono K., Yudasaka M., Bianco A., Miyako E. (2016). Magnetically and Near-Infrared Light-Powered Supramolecular Nanotransporters for the Remote Control of Enzymatic Reactions. Angew. Chem..

[91] Baek S.-K., Makkouk A.R., Krasieva T., Sun C.-H., Madsen S.J., Hirschberg H. (2011). Photothermal treatment of glioma; an in vitro study of macrophage-mediated delivery of gold nanoshells. J. Neurooncol..

[92] Madsen S.J., Baek S.K., Makkouk A.R., Krasieva T., Hirschberg H. (2012). Macrophages as cell-based delivery systems for nanoshells in photothermal therapy. Ann. Biomed. Eng..

[93] Miller M.A., Zheng Y.-R., Gadde S., Pfirschke C., Zope H., Engblom C., Kohler R.H., Iwamoto Y., Yang K.S., Askevold B. (2015). Tumour-associated macrophages act as a slow-release reservoir of nano-therapeutic Pt(IV) pro-drug. Nat. Commun..

[94] Zhang W., Wang M., Tang W., Wen R., Zhou S., Lee C., Wang H., Jiang W., Delahunty I.M., Zhen Z. (2018). Nanoparticle-Laden Macrophages for Tumor-Tropic Drug Delivery. Adv. Mater..

[95] Xu Z., Liu H., Tian H., Yan F. (2020). Real-Time Imaging Tracking of Engineered Macrophages as Ultrasound-Triggered Cell Bombs for Cancer Treatment. Adv. Funct. Mater..

[96] Leonard F., Curtis L.T., Yesantharao P., Tanei T., Alexander J.F., Wu M., Lowengrub J., Liu X., Ferrari M., Yokoi K. (2016). Enhanced performance of macrophage-encapsulated nanoparticle albumin-bound-paclitaxel in hypo-perfused cancer lesions. Nanoscale.

[97] Mantovani A., Allavena P., Marchesi F., Garlanda C. (2022). Macrophages as tools and targets in cancer therapy. Nat. Rev. Drug Discov..

[98] Feng M., Chen J.Y., Weissman-Tsukamoto R., Volkmer J.-P., Ho P.Y., McKenna K.M., Cheshier S., Zhang M., Guo N., Gip P. (2015). Macrophages eat cancer cells using their own calreticulin as a guide: Roles of TLR and Btk. Proc. Natl. Acad. Sci. USA.

[99] Xia Y., Rao L., Yao H., Wang Z., Ning P., Chen X. (2020). Engineering Macrophages for Cancer Immunotherapy and Drug Delivery. Adv. Mater..

[100] Kumari M., Purohit M.P., Pahuja R., Patnaik S., Shukla Y., Kumar P., Gupta K.C. (2019). Pro-inflammatory macrophage polarization enhances the anti-cancer efficacy of self-assembled galactomannan nanoparticles entrapped with hydrazinocurcumin. Drug Deliv. Transl. Res..

[101] Qiang L., Cai Z., Jiang W., Liu J., Tai Z., Li G., Gong C., Gao S., Gao Y. (2019). A novel macrophage-mediated biomimetic delivery system with NIR-triggered release for prostate cancer therapy. J. Nanobiotechnol..

[102] Nguyen V.D., Min H.-K., Kim D.-H., Kim C.-S., Han J., Park J.-O., Choi E. (2020). Macrophage-Mediated Delivery of Multifunctional Nanotherapeutics for Synergistic Chemo–Photothermal Therapy of Solid Tumors. ACS Appl. Mater. Interfaces.

[103] Zhu M.-H., Zhu X.-D., Long M., Lai X., Yuan Y., Huang Y., Zhang L., Gao Y., Shi J., Lu Q. (2023). Metal-Coordinated Adsorption of Nanoparticles to Macrophages for Targeted Cancer Therapy. Adv. Funct. Mater..

[104] Zhang Y., Wang Q., Ma T., Zhu D., Liu T., Lv F. (2020). Tumor targeted combination therapy mediated by functional macrophages under fluorescence imaging guidance. J. Control. Release.

[105] Huang Y., Guan Z., Dai X., Shen Y., Wei Q., Ren L., Jiang J., Xiao Z., Jiang Y., Liu D. (2021). Engineered macrophages as near-infrared light activated drug vectors for chemo-photodynamic therapy of primary and bone metastatic breast cancer. Nat. Commun..

[106] Hou T., Wang T., Mu W., Yang R., Liang S., Zhang Z., Fu S., Gao T., Liu Y., Zhang N. (2020). Nanoparticle-Loaded Polarized-Macrophages for Enhanced Tumor Targeting and Cell-Chemotherapy. Nano-Micro Lett..

[107] Wang Y., Zhang L., Liu Y., Tang L., He J., Sun X., Younis M.H., Cui D., Xiao H., Gao D. (2022). Engineering CpG-ASO-Pt-loaded Macrophages (CAP@M) For Synergistic Chemo-/Gene-/Immuno-Therapy. Adv. Healthc. Mater..

[108] Si J., Shao S., Shen Y., Wang K. (2016). Macrophages as Active Nanocarriers for Targeted Early and Adjuvant Cancer Chemotherapy. Small.

[109] Lee S., Kivimäe S., Dolor A., Szoka F.C. (2016). Macrophage-based cell therapies: The long and winding road. J. Control. Release.

[110] Liang T., Zhang R., Liu X., Ding Q., Wu S., Li C., Lin Y., Ye Y., Zhong Z., Zhou M. (2021). Recent Advances in Macrophage-Mediated Drug Delivery Systems. Int. J. Nanomed..

[111] Li K., Lu L., Xue C., Liu J., He Y., Zhou J., Xia Z., Dai L., Luo Z., Mao Y. (2020). Polarization of tumor-associated macrophage phenotype via porous hollow iron nanoparticles for tumor immunotherapy in vivo. Nanoscale.

[112] Li C.-X., Zhang Y., Dong X., Zhang L., Liu M.-D., Li B., Zhang M.-K., Feng J., Zhang X.-Z. (2019). Artificially Reprogrammed Macrophages as Tumor-Tropic Immunosuppression-Resistant Biologics to Realize Therapeutics Production and Immune Activation. Adv. Mater..

[113] Qiu N., Wang G., Wang J., Zhou Q., Guo M., Wang Y., Hu X., Zhou H., Bai R., You M. (2021). Tumor-Associated Macrophage and Tumor-Cell Dually Transfecting Polyplexes for Efficient Interleukin-12 Cancer Gene Therapy. Adv. Mater..

[114] Rao L., Zhao S.-K., Wen C., Tian R., Lin L., Cai B., Sun Y., Kang F., Yang Z., He L. (2020). Activating Macrophage-Mediated Cancer Immunotherapy by Genetically Edited Nanoparticles. Adv. Mater..

[115] Wang Y., Yu J., Luo Z., Shi Q., Liu G., Wu F., Wang Z., Huang Y., Zhou D. (2021). Engineering Endogenous Tumor-Associated Macrophage-Targeted Biomimetic Nano-RBC to Reprogram Tumor Immunosuppressive Microenvironment for Enhanced Chemo-Immunotherapy. Adv. Mater..

[116] Wu J., Liu L., Chen B., Ou J., Wang F., Gao J., Jiang J., Ye Y., Wang S., Tong F. (2021). Magnetically powered helical hydrogel motor for macrophage delivery. Appl. Mater. Today.

[117] Song X., Fu W., Cheang U.K. (2022). Immunomodulation and delivery of macrophages using nano-smooth drug-loaded magnetic microrobots for dual targeting cancer therapy. iScience.

